# Little long-term change in regional species richness of tropical butterflies over the past 166 years masks turnover in community composition

**DOI:** 10.1098/rspb.2025.1772

**Published:** 2025-10-15

**Authors:** Tiffany L. T. Ki, Colin M. Beale, Blanca Huertas, Jane K. Hill

**Affiliations:** ^1^ Insect Ecology Group, Department of Zoology, University of Cambridge, Cambridge, Cambridgeshire, UK; ^2^ Leverhulme Centre for Anthropocene Biodiversity, Department of Biology, University of York, York, North Yorkshire, UK; ^3^ Science Department, Natural History Museum, London, UK

**Keywords:** community dynamics, tropical biodiversity, museum collections, biodiversity change, dynamic occupancy modelling

## Abstract

Most information on biodiversity changes is from the last few decades despite species responding to environmental changes for centuries. Longer-term information is needed to contextualize whether recent changes reflect longer-term trends. We focus on tropical regions, which are exceptionally biodiverse but contain many species that are currently threatened. We integrate historical and contemporary data from museum collections and online records for 45 butterfly species from Sulawesi (Indonesia) to explore species richness trends over the past 166 years (1857–2022), test whether recent trends mirror longer-term trends and examine whether species that are endemic, forest-dependent and/or host–plant specialists have declined the most. Over the 166-year time period, we found no systematic decline in overall species richness, despite shorter-term multi-decadal changes (positive, stable and negative trends). Recent trends generally did not match longer-term trends. Contrary to expectation, we found long-term increases in some species, particularly those that are non-endemic or open-habitat tolerant, whereas endemic and/or forest-dependent species showed more mixed trends, either stable or declining. We find long-term stability in regional species richness, but this masks composition changes that include more non-endemic and open-habitat species over time. Short-term fluctuations, spanning a few decades, did not reflect longer-term patterns, highlighting challenges in determining robust patterns of biodiversity change.

## 1. Background

There are concerns about global biodiversity changes [1], with some studies reporting considerable declines [2], although others find no systematic pattern of loss in species richness over time [3]. The time span of studies may explain some of this lack of consensus on the severity and direction of biodiversity changes [4]. For example, most biodiversity datasets span only the past 40−50 years, and often much shorter periods, yet species have been responding to centuries of environmental change [3,5]. Examining longer-term datasets would help place recent changes into context and help in understanding if relatively short-term variability (e.g. decadal changes) is consistent with more sustained long-term change over centuries [4,6–8]. Thus, analyses over longer time scales are urgently needed to contextualize current biodiversity changes and to better understand the severity and time span of biodiversity losses.

### (a) Value of natural history collections

The current lack of long-term analyses of species records reflects that most recording schemes to monitor biodiversity have only been instigated relatively recently. However, major sources of historical biodiversity data are held in natural history collections [9], although these are highly underutilized [9,10]. Major natural history collections often span several centuries, providing valuable information and opportunities to explore biodiversity changes over much longer time periods; however, the information held often reflects the collectors’ interests and opportunities to access locations rather than systematic records across space and time. Consequently, there are biases in the coverage of historical information (e.g. spatial, temporal and taxonomic biases in specimens represented), as well as uncertainties in the accuracy of information that can be obtained (e.g. information on collection location [11]). These potential biases pose challenges for interpreting biodiversity change [11], but there are a growing number of analytical techniques being developed to address these uncertainties [12–14]. These techniques have been applied successfully to explore long-term changes in species’ occurrences [12], morphology [13] and phenology [14], highlighting the value of natural history collections in contributing towards a better understanding of long-term biodiversity change. Here, we use these approaches to explore long-term trends in tropical species richness using natural history collections.

### (b) Long-term changes in tropical diversity

Tropical regions support the majority of global diversity, including a large proportion of endemic and range-restricted species [15]. Nonetheless, datasets for monitoring tropical biodiversity are often unavailable or non-existent [5], and so our understanding of changes in tropical biodiversity is very poor [16]. This lack of knowledge is concerning given that tropical ecosystems currently face high rates of forest loss from agricultural expansion [16]; may be especially vulnerable to climate change [17] and land-use change [18], and may be experiencing the greatest current biodiversity losses [19]. The responses of species to environmental changes can vary according to species traits [20,21], with species with restricted geographical ranges, high habitat specificity and/or low population density, traits often typical of tropical species, being likely to have negative trends [22], which may lead to changes in assemblage composition [20,23]. Therefore, analysing longer-term biodiversity data from tropical regions is vital for understanding global biodiversity changes and the consequences for community composition.

### (c) Tropical butterfly richness trends

We focus on butterflies of the island of Sulawesi (Indonesia), a tropical biodiversity hotspot [15] harbouring exceptional levels of endemism [24]. Butterflies are well-represented in recent data depositories (e.g. Global Biodiversity Information Facility, GBIF; https://www.gbif.org) [25], as well as in historical collections [10], and are known to be sensitive to environmental changes, due to their short generation times [26] and being ectothermic [26], making them an excellent taxon for exploring long-term trends. In addition, butterfly traits affect the responses of species to environmental change, such that many tropical butterflies with poor dispersal ability [27], high dependence on forest habitats [28,29], high larval host-plant specificity [28] and low availability of host-plants [27,29] are likely to be particularly vulnerable to anthropogenic changes. We examine Sulawesi’s swallowtail (Papilionidae) and satyrine (Nymphalidae: Satyrinae) butterfly species because they are particularly well represented in collections [24,30] and include both fruit-feeding (Satyrinae) and nectar-feeding (Papilionidae) guilds [24,31–34]. Sulawesi has a high rate of endemism in butterflies, with over 40% of its species being endemic [24]. These butterflies also vary in relation to their dependency on forest and range of host-plants used [24,31–35], allowing examination of differences in long-term trends associated with these traits (see electronic supplementary material, table S1). Integrating data from museum collections with online species records of Sulawesi butterflies thus provides a unique opportunity to examine long-term trends.

### (d) Aim and hypotheses

We investigate long-term trends in Sulawesi Papilionidae and Satyrinae butterflies from 1857 to 2022 (166 years). We use dynamic occupancy modelling to quantify changes in regional species richness, and we test the hypotheses (i) that species richness has declined over time, (ii) that recent changes mirror long-term changes and (iii) that declines have been greatest in species that have restricted ranges, are host-plant specialists and/or dependent on forest habitat.

## 2. Methods

**Figure 1.**
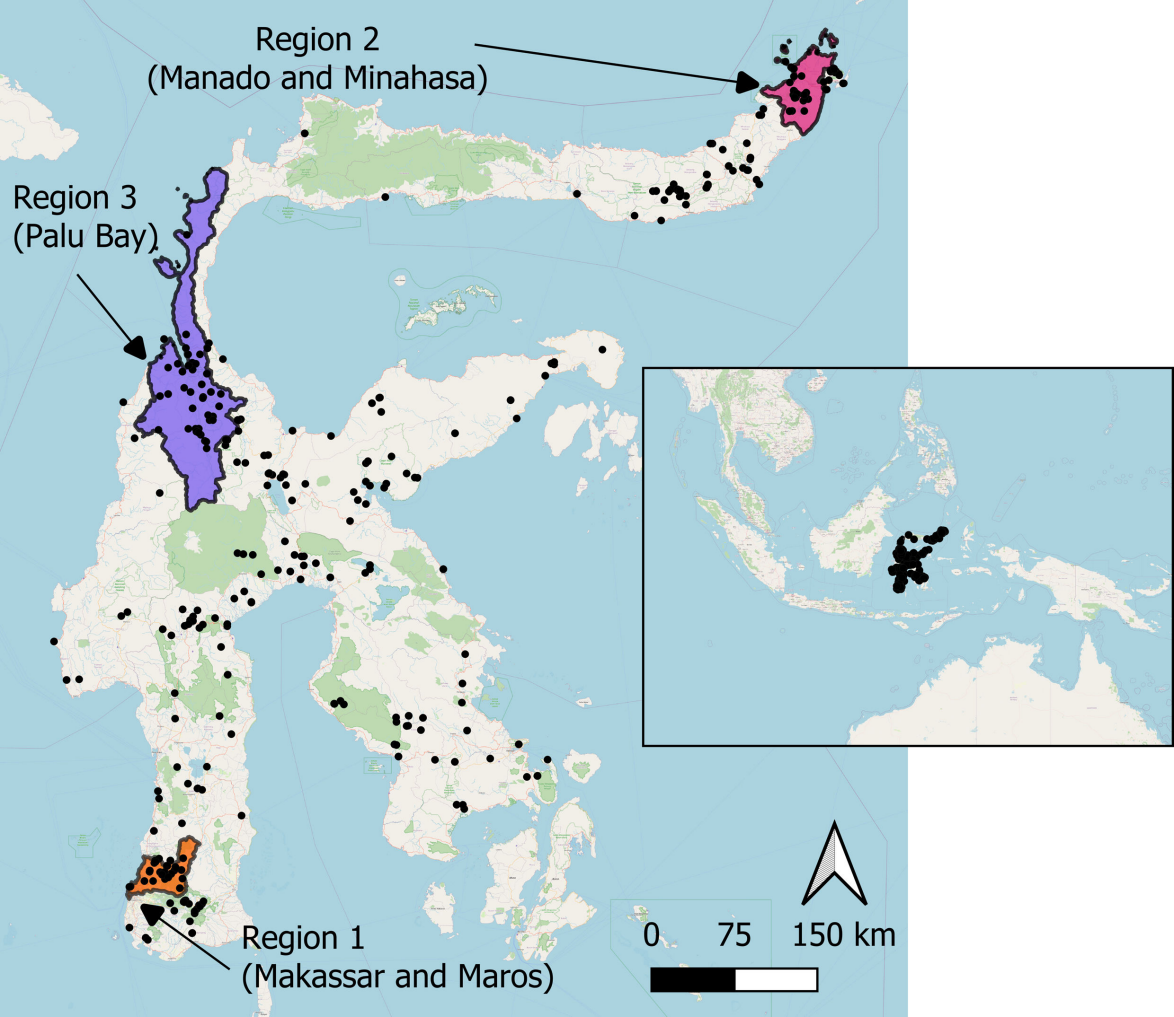
Map of Sulawesi (Indonesia), showing the species records of Papilionidae and Satyrinae butterflies (black circles) and the extent of the three study regions (coloured polygons) examined in this study. The boundaries of the study regions were determined by administrative boundaries (Region 1 = Makassar and Maros; Region 2 = Manado, Minahasa Utara, Minahasa and Tomohon; Region 3 = Palu, Dongala and Sigi; electronic supplementary material, table S2). The inset map shows the location of Sulawesi within Southeast Asia. Map created using QGIS [36]. Where species records were from exactly the same location, the circles will be completely overlaid, resulting in fewer black circles on the map than the total number of records.

Previous analyses have revealed three main regions on Sulawesi with long-term recording [30] (figure 1; electronic supplementary material, table S2), thus we focused our analyses on examining the long-term trends in these three regions (area = 1624–10 723 km^2^).

### (a) Butterfly species records

We obtained 8436 butterfly individual records directly through the digitization of specimens held at the Natural History Museum (UK) and the Zoologische Staatssammlung München (Germany), and indirectly through the GBIF [37,38], which provided specimen records from other museum collections and online records from iNaturalist (see electronic supplementary material, methods for more details). Where point location coordinates were not specified, and depending on information provided, we georeferenced the location of records to centre point coordinates of Indonesian administrative divisions of ‘city’ (area of 163−388 km^2^) or ‘regency’ (1151−1844 km^2^) or finer spatial scales, using the Gazetteer: Celebes [39], Nominatim Open Street Map (https://nominatim.openstreetmap.org), the Fuzzy Gazetteer (https://isodp.hof-university.de/fuzzyg/query) and associated literature, following an established method [30]. We checked all records for inconsistencies with the species checklist of Sulawesi butterflies [24] and for taxonomic accuracy. We excluded records where species identity, year, locality or point coordinates could not be determined. We only included data from the three study regions (figure 1), and we only included species where there were records from at least 2 years (resulting in information on 52 study species; Papilionidae = 27 species, 2277 records, Satyrinae = 25 species, 519 records; electronic supplementary material, figure S1 and table S3). For each year of the study (i.e. each year with one or more butterfly records), we assigned study species as either present (1) if there was one or more records of the species at the given region or else absent (0) in our observation dataset (see §2c on modelling absences over time).

### (b) Butterfly trait information

We categorized species as either endemic or non-endemic to the Sulawesi region following Vane-Wright & de Jong [24]. We categorized species as either forest-dependent or open-habitat tolerant based on their habitat associations [31–33,35]. We categorized species as host-plant specialist (one host-plant family used) or generalist (more than one host-plant family used) based on information about their host-plants [24,31–34]. Host-plant information and habitat descriptions were not available for most Satyrinae, and so we only analysed host-plant specificity and habitat associations in Papilionidae.

### (c) Modelling species richness trends

We used dynamic species occupancy models [40] to model changes in the probability of occurrence of each species in a study region in a particular study year. The dynamic occupancy models are comprised of four parameters: initial occupancy (*ψ*), colonization probability (*ɣ*), extinction probability (*ε*) and detection probability (*p*), which are each modelled as functions of covariates. Initial occupancy is the occupancy state in the first year of the time series. Colonization probability is the probability that a region goes from unoccupied to occupied in year_*n* − 1_ to year_*n*_. Extinction probability is the probability that a region goes from occupied in year_*n* − 1_ to unoccupied in year_*n*_. Detection probability is the probability that a species is detected in a year in which it is present (see electronic supplementary material, methods for more details).

Because recording effort varied over time, between taxa and among regions (electronic supplementary material, figure S1) and so affects the probability of detection of a species in a given year, we computed measures of recording effort based on specimen numbers across all species in a taxon (i.e. Papilionidae or Satyrinae) for each of the three regions from 1853 to 2022 (electronic supplementary material, table S4). We also computed measures of temperature and forest cover because they were likely important influences on butterfly occurrence. We derived regional annual measures of temperature from 1853 to 2022 using the UK Met Office Hadley Centre/Climatic Research Unit global surface temperature data set v. 5 (HADCRUT5) [41] (electronic supplementary material, table S4). We combined historical written descriptions for the earliest periods [42,43] (electronic supplementary material, figure S2), a historical vegetation map from 1950 [44], and a recent forest cover dataset [45], to derive regional annual estimates of forest cover (electronic supplementary material, table S4). For each species, we identified the best covariate to model its probability of colonization or extinction across regions in each year, by fitting four models, where colonization or extinction was modelled as (1) constant, (2) linear over time, or as a function of (3) temperature or (4) forest cover (electronic supplementary material, table S5), and comparing their Akaike information criterion (AIC) values (assuming the best covariate had the model with the lowest AIC value; electronic supplementary material, table S1).

We built dynamic species occupancy models for each butterfly study species (*n* = 52 species), where yearly colonization and extinction probabilities were modelled using the best covariates for each species (electronic supplementary material, table S1) and detection probability was modelled by recording effort, using the R package *unmarked* [46]. There is no information on the true occurrence of each species at the start of the time series (e.g. there were no species checklists completed in 1853), and so we modelled initial occurrence as present (1) for all species and then removed the first year of the species’ probability of occurrence estimates (i.e. 1853) from our statistical analyses. We assessed the goodness-of-fit of each species’s model by comparison of simulated datasets with the observed data (electronic supplementary material, table S6), and we had well-fitting models for 45 species (Papilionidae: 21 species; Satyrinae: 24 species; electronic supplementary material, table S1).

Our dynamic occupancy models estimated the probability of each species’s occurrence (i.e. a value between 0 and 1) in each of the three study regions in each study year between 1853 and 2022 (electronic supplementary material, figure S3 and table S1). We used the outputs from these models to analyse long-term species richness changes between 1857 and 2022 in the three study regions. We also re-ran the analyses, including the 7 species with dynamic occupancy models that had poor goodness-of-fit (Papilionidae: 6 species; Satyrinae: 1 species), to see if our conclusions were affected by their exclusion. Our results were broadly qualitatively consistent, and we report these in the electronic supplementary material, tables S7, S8, and figures S4–S6, and only report results from species with well-fitting models in the main text.

### (d) Richness analysis

We conducted analyses of richness separately for the two butterfly taxa sampled (Papilionidae; Satyrinae) because of differences in recorder effort. For each taxon, we quantified the species richness of each of the three study regions and study years by summing the probabilities of occurrence of all species (from our occupancy model outputs, following recommendations by Calabrese *et al.* [47]). To test the hypothesis that species richness has declined over time, we fitted five models (intercept-only: SR ~ Region; linear additive: SR ~ Year + Region; linear interaction: SR ~Year * Region; nonlinear additive: SR ~ s(Year) + Region; nonlinear interaction: SR ~ s(Year, by = Region) + Region; electronic supplementary material, table S9), with regional species richness as the response variable and region (and year) as the explanatory variables. We considered that the model with the lowest AIC value best characterized species richness change over time (electronic supplementary material, table S9), and the nonlinear models were consistently the best models. To test the hypothesis that recent changes mirrored longer-term changes, we compared long-term trends (i.e. over the full 166 years of records) with more recent trends by fitting the best linear model to the past 10, 25, 50, 75, 100, 125 and 150 years of regional species richness estimates. This choice of recent time spans allowed us to examine how short-term trends relate to long-term trends and when the long-term trend emerges. We then compared the overlap in slope estimates and standard errors of these different time spans with that of the full 166-year data. To test the hypothesis that declines have been greatest in species that have restricted ranges, are host-plant specialists and/or dependent on forest habitat, we first quantified the species richness for a given trait in each region and study year by summing the probabilities of occurrence of species according to whether they were ‘endemic’, ‘non-endemic’, ‘host-plant specialist’, ‘host-plant generalist’, ‘forest-dependent’ or ‘open-habitat tolerant’. We then tested whether species richness declines have been greatest in species that have restricted ranges, are host-plant specialists and/or dependent on forest habitat by fitting three models for each trait group (linear interaction model: SR ~ Year * Region + Year * Trait; simple nonlinear model: SR ~ s(Year, by = Region) + Region; nonlinear interaction model: SR ~ s(Year, by = Region) + Region + s(Year, by = Trait) + Trait; electronic supplementary material, table S10), with regional trait species richness as the response variable. We fitted different models to identify the model that best characterizes the change for each trait across time and identified the best model using AIC values and *F*-tests (electronic supplementary material, table S10; best model with lowest AIC value). Nonlinear interaction models, with the additional interaction of year and trait, were consistently the best models. As part of the process of fitting the nonlinear models, to balance model complexity with overfitting, we first used the automated smoothing parameter selection to identify a suitable degree of flexibility (i.e. knot) and then compared models with more or fewer knots to identify the optimal number of smoothing terms for the function and fixed the number of knots for model comparisons.

We fitted and calculated the predicted annual summed probabilities of occurrence for all our linear models in base R [48] and nonlinear models using the R packages *mgcv* [49] and *tidymv* [50]. We created our figures using the R package *ggplot2* [51]. We also used the R packages *sf* [52] and *tidyverse* [53], and conducted all our analyses in R Studio [54] (v. 2024.4.2.764) using the R v. 4.3.3 [48].

## 3. Results

We analysed changes in regional species richness (45 butterfly species; 21 Papilionidae, 24 Satyrinae) in three regions of Sulawesi (Indonesia, Region 1 = Makassar and Maros; Region 2 = Manado and Minahasa; Region 3 = Palu Bay; figure 1) over 166 years (1857–2022).

### (a) Long-term changes

**Figure 2.**
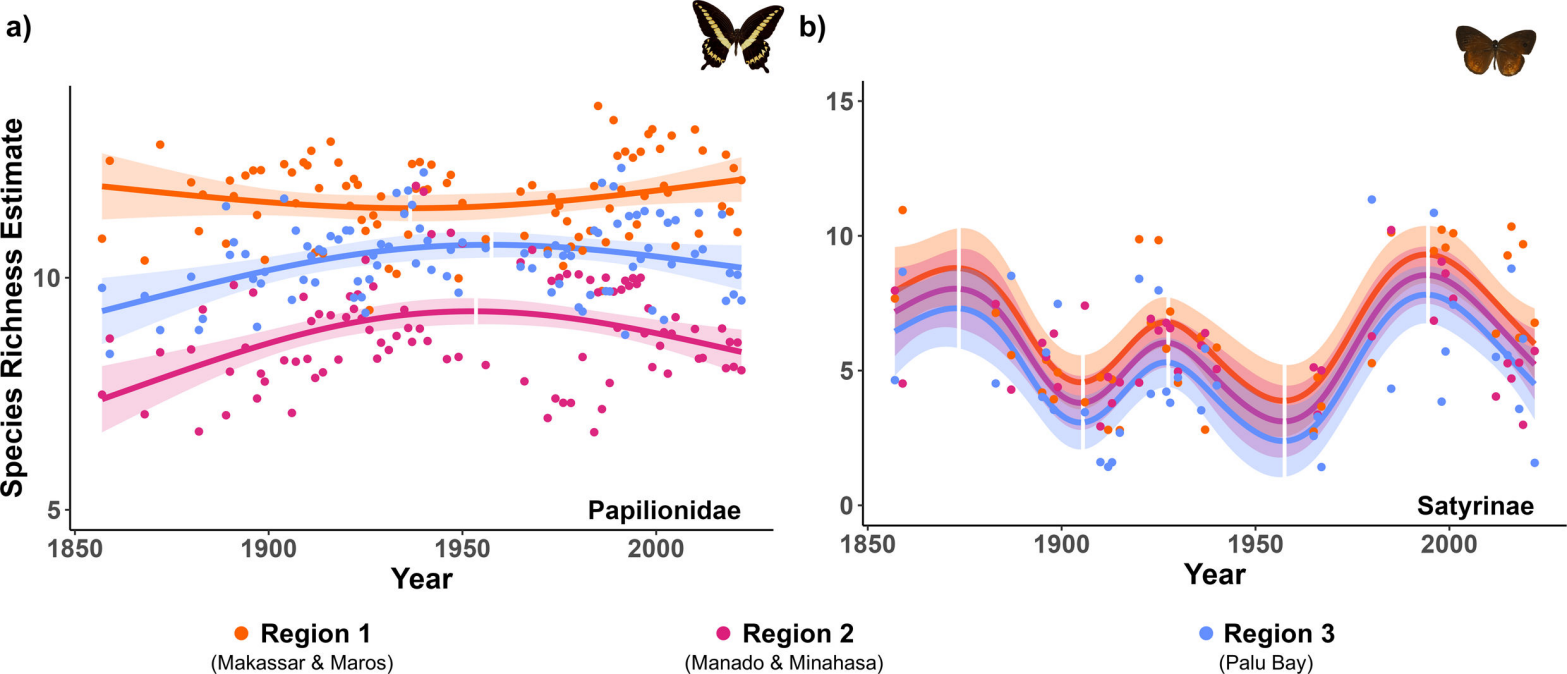
Long-term species richness trends of (a) Papilionidae and (b) Satyrinae butterflies in three regions of Sulawesi (Indonesia). Species richness estimates for each region and study year were computed by the summation of occurrence probabilities across all species in a given region and study year, based on outputs from dynamic occupancy models of 45 species (Papilionidae = 21 species; Satyrinae = 24 species). The points represent the species richness estimate for each of the three regions in each study year (Papilionidae = 94 study years; Satyrinae = 37 study years; see §2 for details). Lines and shading represent the predicted species richness values and 95% confidence intervals from the best fitting models (see electronic supplementary material, table S9). The breaks in the lines and shading indicate points at which the slope of the predicted values (i.e. first derivative) changed signs (see table 2), indicating contrasting shorter-term trends.

Contrary to expectation, we found no consistent decline in species richness over 166 years (figure 2). Across the three regions and two butterfly taxa (i.e. comparing six trends), we found three weakly increasing and three stable trends (table 1). The changes in species richness over time were best characterized by nonlinear models (Papilionidae: *F*_3,273_ = 8.56, *p* < 0.001; Satyrinae: *F*_6,101_ = 9.82, *p* < 0.001; electronic supplementary material, table S9), improving model fits by 3% in Papilionidae and 30.8% in Satyrinae, compared with linear models. This better fit of nonlinear models indicates that the direction and magnitude of species richness changes were not consistent over time in either of the two butterfly taxa, especially Satyrinae.

**Table 1.** Long-term species richness changes in Papilionidae and Satyrinae butterflies in three regions of Sulawesi (Indonesia). Slope estimates and their standard error from the linear interaction model fitted to species richness estimates of each study year (sum of the probabilities of occurrence across all species in a given region; see §2 for details) were multiplied by 10 to give the long-term species richness change (per decade). We determined that trends were increasing (↑) or decreasing (↓), where slope estimates and their standard errors did not overlap with zero, and stable (−), if overlapping.

	Long-term species richness change (per decade)			
	Papilionidae		Satyrinae	
Region 1 (Makassar & Maros)	+0.021 ± 0.025	−	+0.183 ± 0.089	↑
Region 2 (Manado & Minahasa)	+0.032 ± 0.025	↑	+0.023 ± 0.089	−
Region 3 (Palu Bay)	+0.037 ± 0.025	↑	+0.048 ± 0.089	−

### (b) Long-term trends are not consistent with short-term trends or recent data

**Figure 3.**
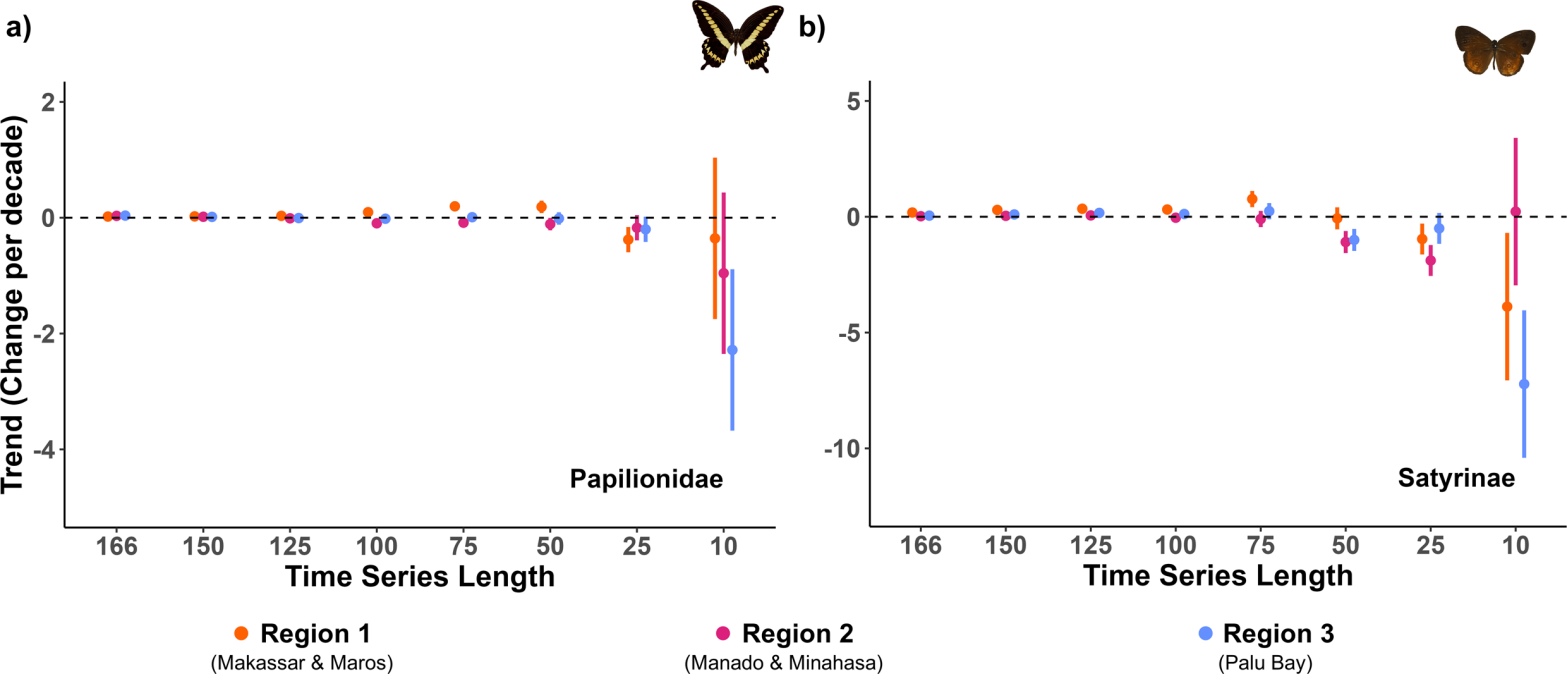
Effect of differing time series length on species richness trends of (a) Papilionidae and (b) Satyrinae butterflies in three regions of Sulawesi (Indonesia). Linear interaction models were fitted to species richness estimates (see §2 for details) for the past 10 (i.e. 2013−2022), 25 (1998−2022), 50 (1973−2022), 75 (1948−2022), 100 (1923−2022), 125 (1898−2022), 150 (1873−2022) and 166 (1857−2022) years of data. The points and error bars represent the model slope estimates and their standard error.

Over the 166-year time period, figure 2 reveals many short-term trends in both taxa that spanned several decades (table 2). Comparing long-term trends (166 years) with recent trends (e.g. past 10 or 25 years), we found qualitatively different conclusions for all three regions and two butterfly taxa (figure 3), indicating that recent trends are not indicative of the longer-term trend. Moreover, there were few consistent patterns within the recent trends, and little evidence for consistent patterns of decline. For example, Satyrinae showed stability in Region 2 based on 10 year trends, while a decline was found in the 25 year trend. In contrast, in Region 3, both the 10 year and 25 year trends showed consistent declines, but the magnitude of decline was much greater in the 10 year trends. Inspection of figure 3 indicates that a consistent long-term trend emerges after about 50 years.

**Table 2.** Summary of the multi-decadal trends in species richness for Papilionidae and Satyrinae butterflies in three regions of Sulawesi (Indonesia). Annual species richness estimate values were predicted from 1857 to 2022 by the best model for Papilionidae (i.e. nonlinear generalized additive model with an interaction between region and year) and Satyrinae (i.e. nonlinear generalized additive model with an additive effect of region and year, electronic supplementary material, table S9). We identified time periods where the slope of the predicted values (i.e. first derivative) is in the same direction (i.e. all positive or all negative) and computed the difference between the species richness indices in the first year and last year of the given period, divided by the number of years in the period. We then multiplied this value by 10 to give the trend (per decade) estimate for each period. Where slope estimates and their standard errors did not overlap with zero, we identified trends as increasing (↑) or decreasing (↓), or if overlapping with zero, trends were considered as stable (−).

	Papilionidae			Satyrinae		
	Time	Trend		Time	Trend	
Region 1 (Makassar & Maros)	1857−1936	−0.059 ± 0.063	−	1857−1873	+0.507 ± 0.933	−
	1937−2022	+0.072 ± 0.045	↑	1874−1905	−1.322 ± 0.397	↓
				1906−1927	+1.012 ± 0.445	↑
				1928−1957	−0.970 ± 0.383	↓
				1958−1994	+1.467 ± 0.329	↑
				1995−2022	−1.185 ± 0.434	↓
Region 2 (Manado & Minahasa)	1857−1953	+0.195 ± 0.052	↑	1857−1873	+0.507 ± 0.933	−
	1954−2022	−0.127 ± 0.056	↓	1874−1905	−1.322 ± 0.397	↓
				1906−1927	+1.012 ± 0.445	↑
				1928−1957	−0.970 ± 0.383	↓
				1958−1994	+1.467 ± 0.329	↑
				1995−2022	−1.185 ± 0.434	↓
Region 3 (Palu Bay)	1857−1957	+0.141 ± 0.050	↑	1857−1873	+0.507 ± 0.933	−
	1958−2022	−0.076 ± 0.059	↓	1874−1905	−1.322 ± 0.397	↓
				1906−1927	+1.012 ± 0.445	↑
				1928−1957	−0.970 ± 0.383	↓
				1958−1994	+1.467 ± 0.329	↑
				1995−2022	−1.185 ± 0.434	↓

### (c) Long-term trends differ according to species traits

**Figure 4.**
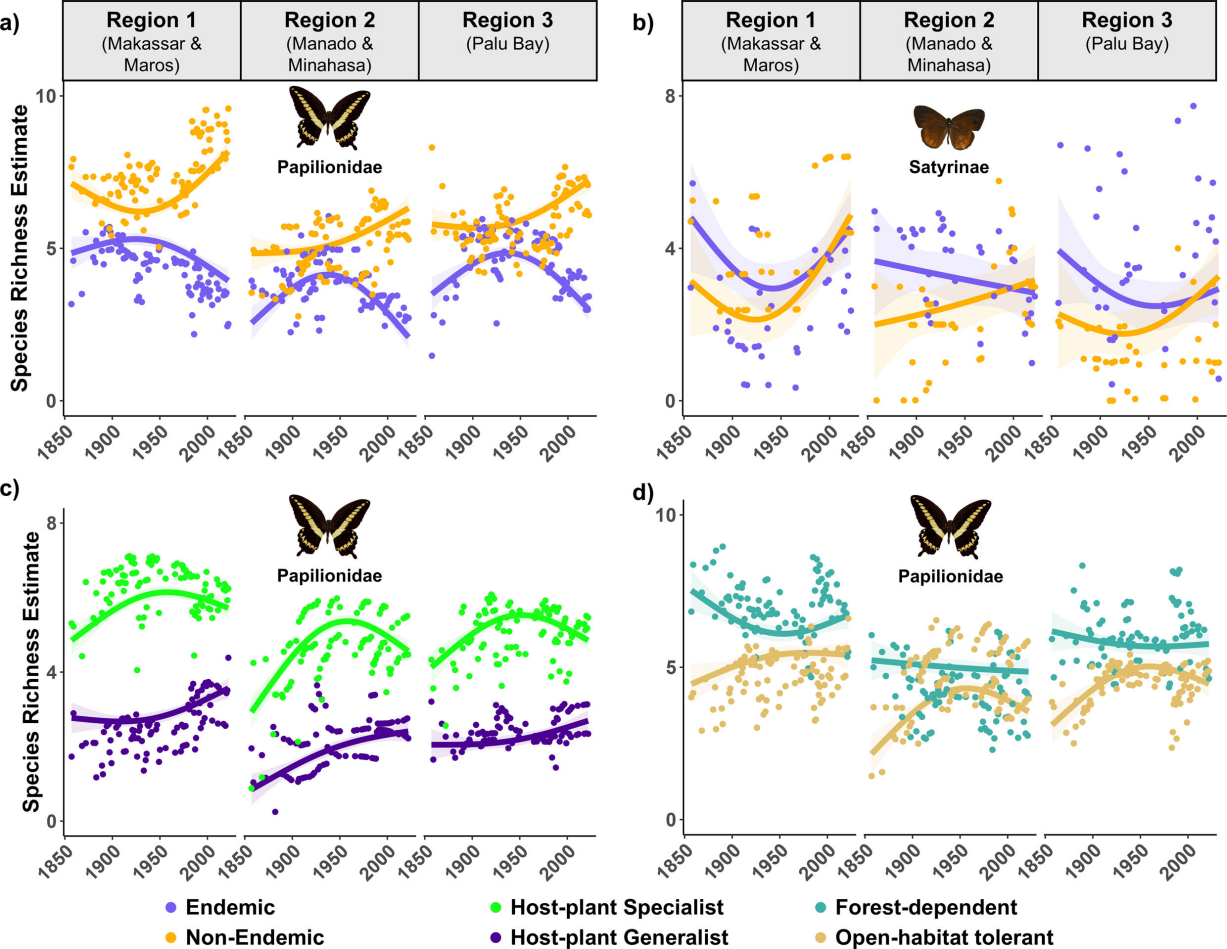
Species richness trends in three regions in Sulawesi (Indonesia) according to endemism in (a) Papilionidae (21 species) and (b) Satyrinae (24 species), (c) host-plant specificity in Papilionidae (16 species), and (d) forest-dependence in Papilionidae (21 species). Data on host-specificity and forest-dependence were only available for Papilionidae. Species richness estimates (points) were computed by summing occurrence probabilities for all species in each trait class in a given region and study year (see §2 for details). The lines and shading represent the predicted values and 95% confidence intervals from the best-fitting model (see electronic supplementary material, table S10).

Surprisingly, we did not find systematic long-term declines in endemic species but instead found mixed responses for endemic species (3 stable and 3 decreasing trends) but all increasing trends for non-endemic species (Papilionidae: *F*_3,552_ = 286.8, *p* < 0.001; figure 4a; Satyrinae: *F*_3,210_ = 5.57, *p* = 0.001; figure 4b; table 3). We only had host-plant (*n* = 16 species) and habitat association (*n* = 21 species) information for Papilionidae, and these revealed long-term species richness increases in all three regions for host-plant generalist (figure 4c; *F*_3, 552_ = 971.0, *p* < 0.001) and open-habitat tolerant (figure 4d; *F*_3,552_ = 70.9, *p* < 0.001) species. Host-plant specialist species showed increasing trends in all three regions, whereas forest-dependent species (one stable and two declining) species showed more mixed responses among regions (table 3). The trends by endemism, host-plant specificity and forest-dependence were best characterized by nonlinear models (electronic supplementary material, table S10), indicating that trends varied in direction and magnitude over the 166-year time period for all traits and in both butterfly taxa (electronic supplementary material, table S11).

**Table 3.** Summary of the long-term species richness trends in three regions (Region 1 = Makassar and Maros, Region 2 = Manado and Minahasa; Region 3 = Palu Bay; figure 1) in Sulawesi (Indonesia) according to species’ endemism (Papilionidae = 21 species; Satyrinae = 24 species); host-plant specificity (Papilionidae = 16 species) and forest-dependence (Papilionidae = 21 species). Slope estimates and their standard error from the linear interaction model fitted to the regional trait species richness estimates (sum of the probabilities of occurrence for all species in a given trait class, region and study year; see §2 for details) are multiplied by 10 to give the long-term trend (per decade).

	Region	Long-term (1857−2022) trend (per decade)			
		**Endemic**		**Non-Endemic**	
Papilionidae	1	−0.073 ± 0.020	↓	+0.094 ± 0.020	↑
	2	−0.067 ± 0.020	↓	+0.100 ± 0.020	↑
	3	−0.065 ± 0.020	↓	+0.102 ± 0.020	↑
Satyrinae	1	+0.030 ± 0.052	−	+0.152 ± 0.052	↑
	2	−0.050 ± 0.052	−	+0.073 ± 0.052	↑
	3	−0.037 ± 0.052	−	+0.085 ± 0.052	↑
		**Host-plant Specialist**		**Host-plant Generalist**	
Papilionidae	1	+0.035 ± 0.015	↑	+0.057 ± 0.015	↑
	2	+0.066 ± 0.015	↑	+0.088 ± 0.015	↑
	3	+0.022 ± 0.015	↑	+0.044 ± 0.015	↑
		**Forest-dependent**		**Open-habitat tolerant**	
Papilionidae	1	−0.028 ± 0.023	↓	+0.048 ± 0.023	↑
	2	−0.022 ± 0.023	−	+0.054 ± 0.023	↑
	3	−0.020 ± 0.023	−	+0.056 ± 0.023	↑

## 4. Discussion

Over a 166-year period (1857−2022), we found no systematic decline in regional species richness. In contrast, we found short-term fluctuations, both increases and decreases, that spanned multiple decades, but these shorter-term recent trends were inconsistent and not indicative of longer-term trends. We found more consistency in trait-based trends, with species that are non-endemics, host-plant generalists and/or tolerant of open habitats showing overall long-term increases, while endemics and/or forest-dependent species showed either stable or declining trends. Thus, the long-term stability in overall species richness masks turnover in community composition, with assemblages containing more non-endemic and open-habitat tolerant species in recent times.

### (a) No systematic declines in richness over 166 years

Our finding of no long-term systematic decline in overall species richness over 166 years is in contrast with other reports of global declines in the diversity and abundance of insects [2] and other taxonomic groups [19]. However, declines are far from ubiquitous [3], and little change as well as increasing trends have also been observed in some tropical insects [55,56]. This lack of consensus in patterns of insect change can be partially explained by differences in biodiversity metrics used in analyses (i.e. measures of biomass, total abundance or richness) which may not be in agreement [3,4]. The lack of consensus may also reflect differences in the spatial scale of analyses, with different biodiversity trends observed when the same data are analysed at different scales [3]. While there is considerable evidence for species richness decline at the global level [3], the evidence suggests no consistent patterns in species richness changes over time at regional and local levels [3,19]. This lack of consensus may be due to differences in responses within and between taxa [55], ecosystems [19] and species’ traits (e.g. dispersal ability [19], range size [22] and specialism [55]), and in our study, we see more consistency when species richness trends are split by trait (figure 4), with endemic species and forest-dependent species showing stable and/or declining trends but non-endemics and open-habitat species showing consistently increasing trends (table 3). Thus, given the known spatial dependency of species richness estimates as well as our study being carried out at a regional/local level, our finding of mixed responses across regions and butterfly taxa is consistent with other studies, as is our finding of more consistent responses when species traits are examined.

### (b) Shorter-term recent trends were inconsistent and not indicative of longer-term trends

Across all three regions and the two butterfly taxa, we found contrasting short-term trends, i.e. different directions and/or magnitudes. Short-term fluctuations are common in natural populations [57] and can be caused by intrinsic factors, such as density dependence [4], as well as by extrinsic factors, such as environmental fluctuations [58]. Insects often display short-term variability in abundance due to their short generation times, high sensitivity to environmental changes and high density dependence [4]. However, the short-term fluctuations we observe are considerably longer in duration than the generation time of insects and span multiple decades. The fact that these fluctuations span several decades suggests they are unlikely to be driven by relatively short-term patterns of climatic variability, such as El-Niño Southern Oscillation cycles (approx. 2–7 years) [59] or the Indian Ocean Dipole (approx. 17 years) [60]. The timings of these short-term fluctuations do not coincide with peaks and troughs in recording effort (electronic supplementary material, figure S1), implying that they are not artefacts of recording. We conclude that the short-term trends we observe are likely to be primarily driven by extrinsic factors, due to the synchronous patterns of change we observed in the three study regions (figure 2) rather than intrinsic factors acting independently in each region, which would lead to more asynchronous patterns of change. We did not find that any of the trait trends varied similarly over time compared with the overall regional trends, indicating that trends are unlikely to be driven by a particular trait group (e.g. host-plant specialists). These trends instead may be due to other factors, such as biotic interactions (e.g. parasites or disease dynamics [61]). We did observe some consistent patterns across regions in host-plant specialist species (peaks around the mid-twentieth century) and endemic swallowtail species (peaks in the first half of the twentieth century, electronic supplementary material, table S11), which may suggest island-wide landscape configuration changes. Major events around this period were the Second World War (1939−1945), Indonesia’s Declaration of Independence (1945), Indonesian National Revolution (1945−1949) and the end of Dutch colonial rule (1949), which may have caused landscape changes.

### (c) Turnover in regional assemblage composition

We found that species that are non-endemic or open-habitat tolerant increased over the study period, compared with endemics and forest-dependent species that did not increase. These results showing that species traits influence trends are consistent with other studies [55], and in our study probably reflect traits with greater tolerance of forest modification [20]. During the 166 years of our study, forest habitats have been modified and converted to other land uses [62,63]. Forest-dependent species are likely to be adversely affected by the reductions in extent and connectivity of remaining forest, especially if they have poor dispersal [20]. In contrast, non-endemic and open-habitat species may be able to move more easily between patches of forest, especially if these species also have high dispersal ability [64], leading to long-term increases. Forest fragmentation leads to increases in edge habitats, that harbour different microclimatic conditions to forest interiors (‘edge effects’ [21]), which may also benefit open-habitat tolerant species [20]. These changes in the occurrence of species according to their traits lead to changes in community composition over time [21]. The loss of lowland forests on Sulawesi over the past 166 years [62,63] has been associated with changes to tropical butterfly assemblages, with increases in species with greater tolerance of forest fragmentation, consistent with findings from other studies on tropical systems (e.g. plants [23], birds [65], insects [28] and vertebrates [66]).

### (d) Detecting species trends from short time series

Lack of long-term data means that most biodiversity trends are estimated from data spanning less than 50 years [4] and often a lot less than that. This raises questions about the length of time series required to reliably estimate species trends. Our results show that short-term trends that span several decades are not representative of longer-term trends (figure 2). We found that butterfly species trends appear to be broadly consistent after about 50 years (figure 3). We show that the length of a time series affects estimates of species trends, and other studies have shown how the length of time series affects the magnitude and/or direction of change [4]. However, short-term trends are often used for identifying species under threat in order to develop interventions to prevent further declines and extinctions [67]. The International Union for Conservation of Nature (IUCN) classifies species’ threats based on population trajectories observed over 3 generations or 10 years (whichever is longer) [67], but our data indicate that information from short-term 10 year trends may be misleading. Our findings echo the caution raised by other studies that these 10 year trends may be capturing short-term variability rather than being indicative of trends [6,7]. Focusing on short-term trends might result in a species being assessed as stable and not under threat, whereas longer time series might reveal long-term decline. Potential solutions are to use both recent (i.e. 10 years) and long-term (e.g. 30+ years) trends to develop threat classifications [6]. In the absence of long-term data, and particularly for species with high inter-annual variability and short generation times (e.g. insects), we suggest that another solution might be to continue to use 10 year trends with caution, supported by frequent reassessments of trends as more data become available. Further research is needed to determine if short-term data are robust for assessing trends in species with lower inter-annual variability than insects (e.g. birds [7]), although taxa with long generation times (e.g. many vertebrate taxa) may have considerable temporal lags in their responses to environmental changes [8].

### (e) Museum collections for extending time series

Estimates of species trends are often restricted to short-term datasets due to lack of longer-term data. In this study, we were able to analyse changes in species richness over a period of 166 years by combining museum specimen data with modern online records. Museum collections contain vast amounts of historical biodiversity data, making them an invaluable resource for extending recent diversity time series. The butterfly museum data we analysed were not collected with a systematic structured design and instead often reflect interests and opportunities available to the collectors [9]. However, our analyses show how recent advances in analytical techniques can be used to analyse these unstructured data to assess much longer-term biodiversity change. The vast stores of historical data held in museum collections have not been easily accessible [9,10], but recent rapid growth in the digitization of museum collections has enabled much greater access to these data [9]. For example, developments in the application of novel technologies to digitize museum specimens have sped up the time-consuming process of data extraction [9]. With these advancements, the availability of historical biodiversity data will continue to grow, providing more data for understanding and interpreting biodiversity changes.

## 5. Conclusion

We found no overall declines in butterfly species richness on Sulawesi over 166 years but found that species that are non-endemic and/or tolerant of open habitats showed long-term increases, while forest-dependent and/or endemic species showed more variability over time, either stable or declining trends. Our results suggest that regional butterfly communities are relatively stable in overall richness but shifting in composition. We also found that short-term recent trends are not representative of long-term trends. Our findings highlight the importance of long-term time series in assessing and contextualizing biodiversity change, and our study demonstrates the value of museum collections as a key resource for extending of biodiversity time series.

## Data Availability

The data used in this study are available on Zenodo [68]. The NHMUK specimen data are available in the NHMUK DataPortal, https://data.nhm.ac.uk/. Supplementary material is available online [69].
